# Statistically Validated Urban Heat Island Risk Indicators for UHI Susceptibility Assessment

**DOI:** 10.3390/ijerph20021172

**Published:** 2023-01-09

**Authors:** Nawhath Thanvisitthpon

**Affiliations:** Department of Architecture, Rajamangala University of Technology Thanyaburi, Pathum Thani 12110, Thailand; nawhath_t@rmutt.ac.th

**Keywords:** risk assessment, UHI indicators, urban management, sustainability

## Abstract

This research proposes a collection of urban heat island (UHI) risk indicators under four UHI risk components: hazard, exposure, sensitivity, and adaptive capacity. There are 46 UHI risk indicators linked to three pillars of sustainability: social equity, economic viability, and environmental protection. In this study, the UHI risk indicators were first validated by experts to determine their relevancy and subsequently applied to randomly sampled dwellers of Thailand’s capital Bangkok. The UHI indicators were further validated with confirmatory factor analysis to determine the factor loadings (0–1) and reliability. Under the hazard component, the percentage of days when the daily minimum temperature is less than the 10th percentile exhibited the highest indicator-level factor loading (0.915). Vehicular traffic was the UHI exposure indicator with the highest factor loading (0.923), and the proportion of green space to build environment was the UHI sensitivity indicator with the highest factor loading (0.910). For the UHI adaptive capacity component, the highest factor loading (0.910) belonged to government policy and action. To effectively mitigate UHI impacts, greater emphasis should be placed on the indicators with highest factor loadings. Essentially, this research is the first to use statistical structural equation modeling to validate UHI indicators.

## 1. Introduction

An urban heat island (UHI) is an urban or metropolitan area with considerably warmer temperatures than its surrounding rural areas as a result of human activities [1]. The conversion of natural land cover into pavement, buildings and other impervious surfaces that absorb and retain heat contributes to the UHI phenomenon [2]. The major impacts of UHIs include increased energy consumption, elevated emissions of air pollutants and greenhouse gases, compromised human health and comfort, and impaired water quality [3,4,5].

UHI risk assessment enables the formulation of public policy and action to mitigate UHI-related impacts in an effective manner and also enhances individual-level UHI adaptive capacity [6,7,8]. According to the intergovernmental panel on climate change, IPCC, a higher adaptive capacity to climate change-induced UHIs reduces socioeconomic losses while improving individual resilience to excessive heat events [9].

**Figure 1:**
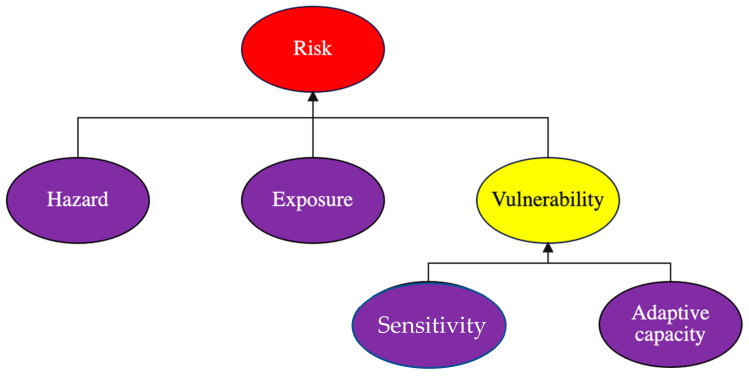
The IPCC’s risk assessment framework [9].

As shown in Figure 1, risk arises from the interaction of hazard, exposure, and vulnerability (i.e., risk = hazard + exposure + vulnerability). Specifically, risk is conceptualized as an internal property of a system that is a function of its current endogenous lack of adaptive capacity to overcome the adverse impact (its sensitivity) of a stressor, and vulnerability is an endogenous characteristic of a system that is determined by its sensitivity and adaptive capacity [9]. In theory, a low adaptive capacity relative to sensitivity (i.e., adverse impacts) results in higher vulnerability and higher risk. As a result, enhancing adaptive capacity reduces vulnerability and subsequent risk.

UHI risk assessment plays a crucial role in limiting ecosystem degradation, social disruptions and economic losses. Specifically, UHI risk assessment provides information about the existing weaknesses of a natural or a socioeconomic system and the plausible causes of the weaknesses. The assessment outcomes enable the formulation of mitigation strategies to deal with or adapt to UHI risks. According to Change [9], climate risk vulnerability reduction is crucial to limiting losses and building resilience to long-term climate change-induced UHI risks.

Specifically, this research relied on the IPCC’s risk assessment framework to develop a collection of UHI risk indicators linked to three pillars of sustainability: social equity, economic viability, and environmental protection. There are 46 UHI risk indicators under four UHI risk components: hazard (consisting of 13 indicators), exposure (10), sensitivity (12), and adaptive capacity (11). 

For the hazard component, the UHI indicator selection is based on 13 future temperature-based extreme climatic indexes, where six indexes are associated with temperature (HT1–HT6) and the remaining seven are associated with duration (HD1–HD7) (Table 1). Additionally, the UHI indicators under the exposure component are associated with the three pillars of sustainability: social equity (ES1–ES4), economic viability (EE1–EE3), and environmental protection (EV1–EV3) (Table 2).

The UHI indicators under the sensitivity component are related to the three pillars of sustainability: social equity (SS1–SS5), economic viability (SE1–SE3), and environmental protection (SV1–SV4) (Table 3). Likewise, for the adaptive capacity component, the UHI indicator selection is based on the three pillars of sustainability: social equity (AS1–AS4), economic viability (AE1–AE4), and environmental protection (AV1–AV3) (Table 4).

These 46 UHI risk indicators were first validated by experts to determine their relevancy using a relevant/irrelevant questionnaire (Supplementary Materials S1), and they were subsequently applied to a sample of Bangkok residents using an agree/disagree questionnaire (Supplementary Materials S2). Based on the agree/disagree survey data, the UHI indicators were further validated with confirmatory factor analysis (CFA) to determine the factor loadings (0–1).

By definition, CFA-based factor loading is the correlation coefficient for the variable (i.e., UHI indicators) and factor (i.e., UHI phenomenon). In structural equation modeling, a factor loading of 0.6 or higher indicates that the factor (i.e., UHI phenomenon) is significantly influenced by the variable (i.e., UHI indicators). In this research, the factor loadings were calculated by using the AMOS (analysis of a moment structures) statistical software. AMOS is an added SPSS module and is used for structural equation modeling, path analysis, and confirmatory factor analysis. In addition, the correlations (R^2^) between UHI indicators were determined with a structural equation model (SEM). Essentially, the proposed CFA-validated UHI indicators could be deployed to effectively assess the susceptibility of an urban area to UHI risks. Moreover, the proposed UHI indicators are applicable to urbanized areas of varying size (e.g., first-, second-, and third-tier cities) to assess the climate change-induced UHI susceptibility and future impacts. Furthermore, this research is the first to apply the SEM technique to statistically validate UHI indicators.

## 2. Research Methodology

In the development of UHI risk indicators, this research first reviewed the literature and publications related to the IPCC’s risk assessment framework (Figure 1). The UHI risk indicators considered in this study are linked to the three pillars of sustainability (i.e., social equity, economic viability, and environmental protection). There are 46 UHI risk indicators associated with four UHI risk components: hazard (consisting of 13 indicators), exposure (10), sensitivity (12), and adaptive capacity (11) [10,11,12,13,14,15].

The hazard indicators were obtained from the open-source RClimDex1.1 program (http://etccdi.pacificclimate.org/software.shtml, accessed on 12 January 2022), which provides a friendly graphical user interface to compute all core climate change indices defined by the joint CCl/WCRP/JCOMM Expert Team on Climate Change Detection and Indices (ETCCDI). For the hazard component, the indicator selection was based on 13 future temperature-based extreme climatic indexes, where six indexes are associated with temperature (HT1–HT6) and the remaining seven are associated with duration (HD1–HD7) (Table 1). 

Meanwhile, the exposure (10), sensitivity (12), and adaptive capacity indicators (11) were obtained from the literature review, as indicated in the references column in Table 2, Table 3 and Table 4, respectively. For the exposure component, the UHI indicator selection was based on the three pillars of sustainability: social equity (ES1–ES4), economic viability (EE1–EE3), and environmental protection (EV1–EV3) (Table 2). Likewise, the UHI indicators for the sensitivity component are related to the three pillars of sustainability: social equity (SS1–SS5), economic viability (SE1–SE3), and environmental protection (SV1–SV4) (Table 3). For the adaptive capacity component, the UHI indicator selection was based on the three pillars of sustainability: social equity (AS1–AS4), economic viability (AE1–AE4), and environmental protection (AV1–AV3) (Table 4).

The 46 UHI risk indicators were converted into a relevant/irrelevant questionnaire. To evaluate the relevancy of the UHI risk indicators, a total of 32 experts in the fields of architecture, climate change, urban environment and urban planning were randomly selected from Google Scholar (https://scholar.google.com/, accessed on 12 January 2022)*,* and copies of the relevant/irrelevant questionnaire were emailed to them. There were seven responses from a total of 32 experts, and a number of expert responses ≥3 is acceptable. 

The expert-validated UHI indicators were subsequently converted into an agree/disagree questionnaire based on a 10-point Likert scale, where 1 denotes strongly disagree and 10 denotes strongly agree. According to Bayraktar and Tatoglu [16], Ismail Salaheldin [17], Thanvisitthpon and Shrestha [18], an ordinal scale can be used with agree/disagree questions. 

The agree/disagree questionnaire was applied to a random sample of individuals to further validate the 46 UHI risk indicators. The sample size was 400 individuals, calculated with the following equation where *n* is the sample size, *N* is the number of people in Bangkok (10.72 million), and e is the sampling error (0.05) (Yamane [19]).

*N* = *N*/1 + *N* (*e*)^2^ (1)

Due to incomplete data, the actual sample size (i.e., questionnaire respondents) was 398 individuals who resided in Thailand’s capital Bangkok. In the data collection, the 398 questionnaire respondents were randomly recruited to answer the agree/disagree questionnaire, and the responses were input into the structural equation model (SEM) analysis. 

Prior to SEM analysis, the Kolmogorov–Smirnov test was applied to the data from the 398 questionnaire respondents, with the null hypothesis (H_0_) being that the data are normally distributed [20]. H_0_ is accepted if the observed statistic is greater than the critical value (α > 0.05), indicating that the data are normally distributed [21,22,23,24].

In the SEM analysis, the 46 UHI risk indicators and four UHI risk components were validated with confirmatory factor analysis (CFA) to determine the factor loadings and reliability of the UHI risk indicators and dimensions. Indicator- and dimension-level factor loadings (0–1) were used to indicate the degree of relevancy of UHI risk indicators and dimensions. The reliability of the UHI risk indicators and dimensions were measured with indicator-level reliability (R^2^) and dimension-level composite reliability (CR).

Since the SEM technique requires a large quantitative dataset or sample size (i.e., 398 respondents in this research) (Dawson and Peppe [25], Thanvisitthpon [26]), its main advantage lies in the validity of the association, as indicated by the factor loadings between the UHI risk indicators and the UHI phenomenon. Additionally, SEM-based CFA relies on the means and variance–covariance matrix instead of on the correlation matrix, thereby minimizing both nonuniform and uniform bias [26]. Furthermore, SEM-based CFA is used when there is an array of variables (i.e., UHI indicators) measuring more than one dimension (i.e., four UHI components—hazard, exposure, sensitivity, and adaptive capacity). However, the limitations of SEM-based CFA include the necessity of a large sample size (despite higher reliability) and the required training and skills to use the AMOS statistical software.

## 3. Results and Discussion

### 3.1. UHI Indicator Relevancy Assessment

In the assessment of the UHI risk indicators, the randomly chosen experts (i.e., 32 experts) were asked to evaluate the relevancy of the 46 UHI risk indicators under four UHI risk components (i.e., hazard, exposure, sensitivity and adaptive capacity) on a relevant/irrelevant scale of −1 to 1, where −1, 0, and 1 denote irrelevant, uncertain, and relevant, respectively. Seven experts responded the relevant/irrelevant questionnaire: two architects, one climate change scientist, two urban planners, and two urban environmentalists. 

Table 1, Table 2, Table 3 and Table 4 tabulate the UHI indicators, definitions, and item-objective congruence (IOC) indexes under the four UHI risk components of hazard, exposure, sensitivity, and adaptive capacity, respectively. The IOC index was used as the basis for screening the item quality, whereby experts were asked to determine the relevancy score of each item (−1, 0, or 1) given that IOC > 0.6 is acceptable [27,28].

**Table 1:** The UHI hazard indicators, definitions and expert-validated IOC indexes.

Hazard Indicators (13)				
Dimension	ID	Indicators	Definition/Detail	IOC
Temperature	HT1	CSDI	Cold spell duration index (CSDI) is defined as annual or seasonal count of days with at least 6 consecutive days when the daily minimum temperature falls below the 10th percentile in the 5-day calendar window for a 30-year period.	0.692
	HT2	DTR	Daily temperature range	0.692
	HT3	TXx	Monthly maximum value of the daily maximum temperature	0.846
	HT4	TNx	Monthly maximum value of the daily minimum temperature	0.769
	HT5	TXn	Monthly minimum value of the daily maximum temperature	0.769
	HT6	TNn	Monthly minimum value of the daily minimum temperature	0.769
Duration	HD1	SU	Number of summer days	0.692
	HD2	TR	Number of tropical nights	0.769
	HD3	TX90p	Percentage of days when the daily maximum temperature is greater than the 90th percentile	0.692
	HD4	TX10p	Percentage of days when the daily maximum temperature is less than the 10th percentile	0.769
	HD5	TN90p	Percentage of days when the daily minimum temperature is greater than the 90th percentile	0.846
	HD6	TN10p	Percentage of days when the daily minimum temperature is less than the 10th percentile (TN10p)	0.923
	HD7	WSDI	Warm spell duration index (WSDI) is defined as annual or seasonal count of days with at least 6 consecutive days when the daily maximum temperature exceeds the 90th percentile in the 5-day calendar window for a 30-year period.	0.769

**Table 2:** The UHI exposure indicators, definitions and expert-validated IOC indexes.

Exposure Indicators (10)					
Dimension	ID	Indicators	Definition/Detail	IOC	References
Social	ES1	Population size	A metropolis with a population of over one million has an average annual temperature 1–3 °C higher than surrounding rural areas.	0.615	[29,30,31]
	ES2	Electricity consumption	Electricity use is positively correlated with the UHI phenomenon. Specifically, electricity consumption and land surface temperature are strongly correlated with an R^2^ of 70–90%.	1.000	[32,33,34]
	ES3	Compromised human health and comfort	The UHI phenomenon contributes to heat-related deaths and illnesses such as general discomfort, respiratory difficulties, heat cramps, heat exhaustion, and non-fatal heat stroke. Sensitive populations are particularly at risk during excessive heat events, including the elderly, young children, those working outdoors, and those with preexisting health conditions.	0.615	[35,36,37,38]
	ES4	Vehicular traffic	Vehicular traffic is the aggregation of vehicles coming and going in a particular locality. Vehicular traffic is positively correlated with UHI intensity and air pollution.	1.000	[32,33,34,35,36,37,38,39]
Economic	EE1	Higher cost of living in urban areas	The UHI phenomenon is significantly positively correlated with population size (*p* ≤ 0.01), economic size (*p* ≤ 0.01), and urbanization (*p* ≤ 0.05).	0.692	[13,40]
	EE2	Land use type	The conversion of agricultural areas into commercial and industrial areas contributes to the UHI phenomenon. Urbanization expansion and increased human activities also worsen the UHI phenomenon.	0.846	[41,42,43]
	EE3	Urban economic growth	Urbanization and economic growth contribute to population growth and increased economic activities, exacerbating the UHI phenomenon.	0.769	[13,40,44,45]
Environment	EV1	Land surface temperature	Land surface temperatures vary in tandem with ambient temperatures. Increasing urban green space lowers land surface temperatures, which consequently reduces ambient air temperatures.	0.846	[46,47]
	EV2	Impervious surface area	Impervious cover is any type of human-made surface that cannot effectively absorb or infiltrate rainfall, such as driveways, paved roads, parking lots, rooftops, and sidewalks. The expansion of an impervious surface area contributes to worsening UHI problems.	0.846	[32,45,47,48,49,50]
	EV3	Pervious surface area	A pervious surface is land not covered by buildings or other man-made infrastructure, thus allowing rainwater to percolate into the soil to filter out pollutants and recharge the groundwater. Pervious surface coverage and the UHI phenomenon are inversely related.	1.000	[32,46,47,48]

**Table 3:** The UHI sensitivity indicators, definitions and expert-validated IOC indexes.

Sensitivity Indicators (12)					
Components	ID	Indicators	Definition/Detail	IOC	References
Social	SS1	Population density and growth	Population density and growth is closely linked to human activities and the UHI phenomenon. A larger population contributes to higher greenhouse gas emissions as a result of increased vehicular traffic and electricity consumption, giving rise to dramatic temperature increases.	0.769	[31,32,44,51]
	SS2	Built environment	The built environment, including buildings, public utilities and infrastructure, touches all aspects of human life. Specifically, the conversion of green area into built environment contributes to the UHI phenomenon and the situation worsens as the conversion intensifies.	0.769	[32,41,42]
	SS3	Total energy consumption	Total energy consumption is the sum of energy used for electricity, transport and heating. Total energy consumption is positively correlated with human activities, which consequently contribute to UHI effects.	1.000	[32,52,53,54,55]
	SS4	Prevalence of noncommunicable diseases	Noncommunicable diseases (NCDs) are of long duration and are the result of a combination of genetic, physiological, environmental and behavioral factors. Examples of NCDs are cardiovascular disease, diabetes, high blood pressure, and obesity. Individuals with NCDs or preexisting health conditions are highly susceptible to UHI-related excessive heat events.	0.692	[37,38,56]
	SS5	Traffic congestion	Increased vehicular traffic and road congestion exacerbates the UHI situation and air pollution.	1.000	[32]
Economic	SE1	Low economic status	Individuals with low income are highly susceptible to UHI impacts. Specifically, low-income households have inadequate income or wealth to mitigate the negative impacts of UHI-induced excessive heat events.	0.615	[13,40,57]
	SE2	Monthly electricity expenditure	Monthly household electricity cost is in direct proportion to electricity consumption. Higher electricity consumption consequently aggravates the UHI situation.	1.000	[32,58]
	SE3	Monthly energy spending	Higher monthly household energy (motor fuels) spending is in direct proportion to increased vehicle use, which consequently contributes to the UHI phenomenon and air pollution.	0.615	[32,58]
Environment	SV1	Proportion of green space to built environment	Green space provides cool and shaded areas while moderating ambient temperatures. The proportion of green space to the man-made built environment is inversely correlated with the UHI phenomenon.	1.000	[32,42,45,59]
	SV2	Atmosphericpollution	Anthropogenic atmospheric pollutants contribute to rising temperatures and the UHI phenomenon. UHI-causing atmospheric pollutants include CO, NO_2_, O_3_, PM2.5, and PM10.	0.846	[32,60]
	SV3	Proportion of water body to built environment	Temperatures in and near water bodies are significantly lower than those covered by the built environment. Additionally, the proportion of water bodies to man-made built environments is inversely correlated with the UHI phenomenon.	1.000	[32,41,42]
	SV4	Building density	Building density (measured by dwelling units per km^2^) determines how crowded or built-up an area of land is. An area with a high building density exhibits a high UHI intensity.	1.000	[32,61]

**Table 4:** The UHI adaptive capacity indicators, definitions and expert-validated IOC indexes.

Adaptive Capacity Indicators (11)					
Components	ID	Indicators	Definition/Detail	IOC	References
Social	AS1	Public understanding	The public understanding of the UHI phenomenon and its impacts enhances adaptive capacity to excessive heat events or UHI-induced temperature increases.	0.846	[62,63,64]
	AS2	Public awareness	A greater public awareness of the UHI phenomenon and its risks contributes to increased UHI adaptive capacity and resilience.	0.846	[62,63,64,65,66]
	AS3	Multi-agency collaboration	Multi-agency collaboration on the dissemination of information and UHI impact mitigation enhances adaptive capacity to abrupt and dramatic temperature increases.	0.846	[63,65,67,68]
	AS4	Governmentpolicy and action	Governments (i.e., local, regional and national levels) should establish UHI mitigation policies and measures to enhance adaptive capacity to UHI-induced excessive heat events.	1.000	[32,42,59,63,69]
Economic	AE1	Green budgeting	Green budgeting refers to the adoption of budgetary tools to achieve environmental and climate goals. Higher green budget allocation enhances adaptive capacity to the UHI phenomenon.	0.846	[8,32,63,70]
	AE2	Private sector participation in green economy	A green economy is an economy that aims to reduce environmental risks and ecological scarcities while achieving sustainable development without degrading the environment. Specifically, the active participation of the private sector in the green economy enhances adaptive capacity to UHI-related dramatic temperature increases.	0.846	[8,32,63,71]
	AE3	Access to climate controlappliances	Access to climate control appliances, e.g., electric fans, cooling fans, and air conditioners, increases adaptive capacity to UHI-induced excessive heat events.	0.846	[72,73]
	AE4	Household medical budget	The size of a household’s medical budget is positively correlated with adaptive capacity to UHI-related illnesses.	0.615	[37,74,75]
Environment	AV1	Proportion of green space to living space	Green space is land that is partly or completely covered with grass, trees, shrubs, or other vegetation. Meanwhile, living space refers to areas in a dwelling unit that are livable spaces. A higher proportion of green space to living space improves adaptive capacity to UHI-induced temperature increases.	0.846	[8,32,76]
	AV2	Adequacy of urban green space	Urban green space refers to open-space areas reserved for parks and recreational activities. Adequate urban green space improves adaptive capacity to UHI-related abrupt temperature increases.	0.846	[8,32,41,42]
	AV3	Resilience to air pollution and UHI	Individual resilience to atmospheric pollutants and extreme heat events enhances adaptive capacity to UHI-related illnesses and deaths.	0.846	[32,77]

### 3.2. Confirmatory Factor Analysis of UHI Hazard Indicators

Two dimensions constitute the UHI hazard component: temperature and duration. The temperature dimension consists of six UHI hazard indicators, and the duration dimension consists of seven indicators. The UHI hazard indicators (13 indicators) were validated with confirmatory factor analysis (CFA) to determine their factor loadings and reliability (first-order) and their dimensions (second-order). Structural equation modeling (SEM) was used to analyze the correlations between the UHI indicators.

**Figure 2:**
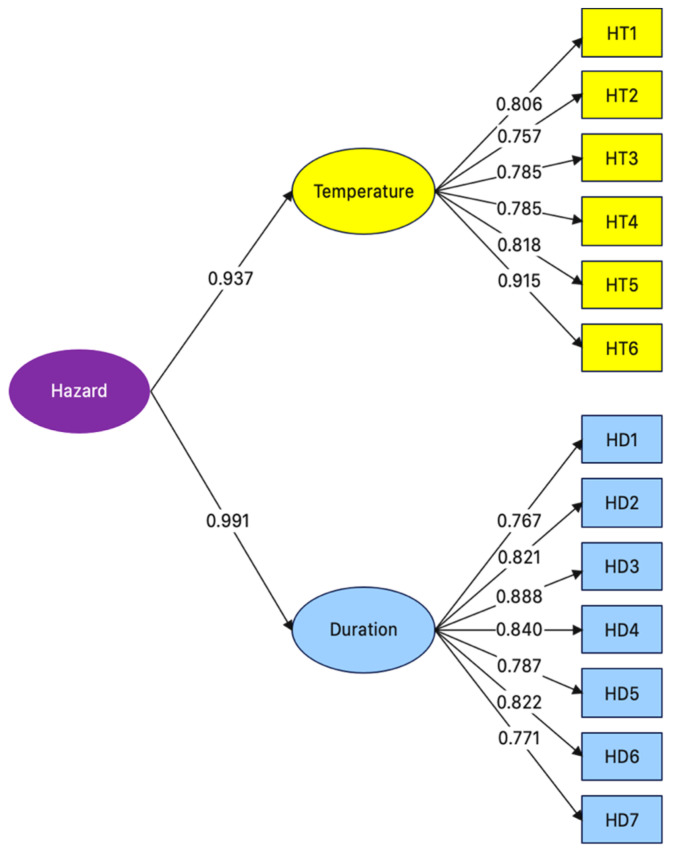
Structural equation model and the factor loadings of the UHI hazard indicators and dimensions.

Figure 2 shows the structural equation model and the factor loadings of the UHI hazard indicators, where chi-squared = 4413.707, degree of freedom (df) = 9, *p*-value = 0.114, root mean square residue (RMR) = 0.050, root mean square error of approximation (RMSEA) = 0.038, goodness of fit index (GFI) = 0.995, adjusted goodness of fit index (AGFI) = 0.945, normed fit index (NFI) = 0.997, and confirmatory fit index (CFI) = 0.999. According to Baumgartner and Homburg [78], Gatignon [79] and Hooper and Coughlan [80], GFI, AGFI, NFI and CFI should be close to 1, while RMSEA and RMR should not exceed 0.050.

Table 5 tabulates the first- and second-order factor loadings of the UHI hazard indicators and dimensions. The factor loadings of the HT1–HT6 and HD1–HD7 indicators were 0.757–0.915 (R^2^ = 0.617–0.837) and 0.764–0.888 (R^2^ = 0.583–0.788), respectively, while those of the temperature and duration dimensions (b) were 0.937 and 0.991, respectively. In comparison, the factor loading of duration was slightly higher, suggesting that the duration dimension plays a more significant role in the UHI hazard component, consistent with [81,82]. According to Kim and Mueller [83], a factor loading of >0.3 is statistically significant. The composite reliability (CR) of the temperature and duration dimensions was 0.921 and 0.932, respectively, with corresponding average variance extracted (AVE) values of 0.660 and 0.663. According to Fornell and Larcker [84], a CFA construct is valid if CR > 0.6 or AVE > 0.5.

On the indicator level, the highest factor loading (0.915) belonged to HT6 (percentage of days when the daily minimum temperature is less than the 10th percentile, TN10). According to Shrestha and Bajracharya [85], Zhang and Aguilar [86], Wong and Mok [87], Manalo and Matsumoto [88], and Dong and Xu [89], TN10 is a key indicator of UHI trends and is commonly used in climate change-induced UHI trend analysis. 

### 3.3. Confirmatory Factor Analysis of UHI Exposure Indicators

The UHI exposure component comprises three dimensions: social, economic and environment. The three exposure dimensions are linked to three pillars of sustainability, i.e., social equity, economic viability, and environmental protection. Specifically, the social, economic and environment dimensions consist of four, three and three UHI exposure indicators, respectively. The UHI exposure indicators (10 indicators) were validated with CFA to determine their factor loadings and reliability (first-order) and their dimensions (second-order), and SEM was used to analyze the correlations between the UHI indicators.

**Figure 3:**
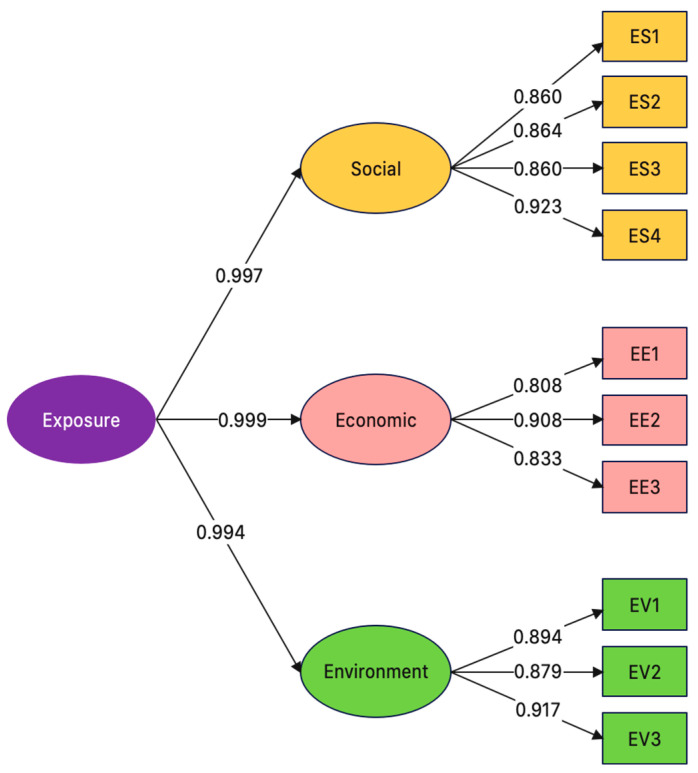
Structural equation model and the factor loadings of UHI exposure indicators and dimensions.

Figure 3 shows the structural equation model of the UHI exposure indicators, where chi-squared = 5925.738, df = 45, *p* = 0.112, RMR = 0.028, RMSEA = 0.040, GFI = 0.996, AGFI = 0.960, NFI = 0.998 and CFI = 0.999. 

Table 6 presents the first- and second-order factor loadings of the UHI exposure indicators and dimensions. The factor loadings of ES1–ES4, EE1–EE3 and EV1–EV3 were 0.860–0.923 (R^2^ = 0.739–0.853), 0.808–0.908 (R^2^ = 0.683–0.825), and 0.879–0.917 (R^2^ = 0.772–0.840), respectively, while those of the social, economic and environment dimensions (*b*) were 0.997, 0.999, and 0.994, respectively; a factor loading >0.3 is statistically significant (Kim and Mueller [83]. The CR values of the social, economic, and environment dimensions were 0.930, 0.887, and 0.925, respectively, with corresponding AVE values of 0.770, 0.724, and 0.804. According to Fornell and Larcker [84], a CFA construct is valid if CR >0.6 or AVE > 0.5.

The highest indicator-level factor loading (0.923) belonged to ES4 (vehicular traffic). Vehicular traffic is the aggregation of vehicles coming and going in a particular locality and it was positively correlated with the UHI intensity and air pollution. According to Hoehne and Chester [90], vehicular traffic and transportation network design play crucial roles in the UHI phenomenon. Hart and Sailor [91] reported that road surface temperatures during weekdays are 2 °C higher than during the weekend. This finding could be attributed to the higher level of vehicular traffic during weekdays. Sailor and Lu [92] reported that heat from vehicles in six U.S. cities accounted for 47%–62% of total heat emissions.

Specifically, increased vehicular traffic contributes to higher greenhouse gas emissions, thereby worsening the UHI situation. As a result, individual-, community-, and institutional-level adaptation measures have been proposed to mitigate the impacts of UHIs [93,94,95,96,97,98].

The second highest factor loading (0.917) belonged to EV3 (pervious surface area). By definition, a pervious surface is land not covered by buildings or other man-made infrastructure, thus allowing rainwater to percolate into the soil to filter out pollutants and recharge the groundwater. Pervious surface coverage and the UHI phenomenon were found to be inversely related.

The conversion of pervious to impervious surfaces as a result of economic development and urbanization expansion causes temperatures in urban areas to be significantly warmer than surrounding rural areas [99,100]. Impervious surface expansion raises urban land surface temperatures (Haselbach and Boyer [100], Shuster and Bonta [101], Gorsevski and Taha [102], Taha [103], Sen and Roesler [104], and Cao and Li [105]). 

### 3.4. Confirmatory Factor Analysis of UHI Sensitivity Indicators

**Figure 4:**
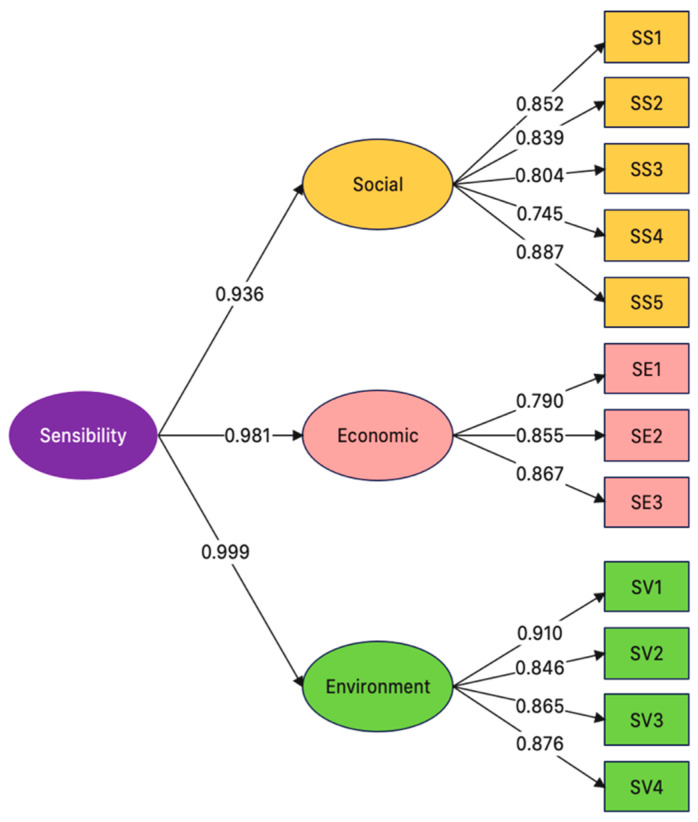
Structural equation model and the factor loadings of UHI sensitivity indicators and dimensions.

The UHI sensitivity component comprises three dimensions linked to three pillars of sustainability, i.e., social equity, economic viability, and environmental protection. The social, economic and environment dimensions of the UHI sensitivity component consist of five, three, and four UHI sensitivity indicators, respectively. The 12 UHI sensitivity indicators were validated with CFA to determine their factor loadings and reliability (first-order) and their dimensions (second-order), and SEM was used to analyze the relationships between the UHI indicators. Figure 4 shows the SEM of the UHI sensitivity indicators, where chi-squared = 5026.587, df = 66, *p* = 0.197, RMR = 0.040, RMSEA = 0.027, GFI = 0.992, AGFI = 0.957, NFI = 0.996 and CFI = 0.999. 

Table 7 tabulates the first- and second-order factor loadings of the UHI sensitivity indicators and dimensions. The factor loadings of SS1–SS5, SE1–SE3 and SV1–SV4 were 0.745–0.887 (R^2^ = 0.500–0.727), 0.790–0.867 (R^2^ = 0.582–0.751), and 0.846–0.910 (R^2^ = 0.721–0.827), respectively, while those of the social, economic and environment dimensions (*b*) were 0.936, 0.981, and 0.999, respectively; a factor loading > 0.3 is statistically significant (Kim and Mueller [83]. The CR values of the social, economic, and environment dimensions were 0.915, 0.876, and 0.982, respectively, with corresponding AVE values of 0.684, 0.702, and 0.765. According to Fornell and Larcker [84], a CFA construct is valid if CR >0.6 or AVE > 0.5.

The proportion of green space to a built environment indicator (SV1) had the highest factor loading (0.910). Urban green space provides cool and shaded areas while moderating ambient temperatures [106]. Urban green spaces, including rooftop and vertical gardens, help tackle UHI problems [107,108]. Specifically, UHI impacts are inversely correlated with the proportion of urban green space to a built environment [109,110,111,112]. 

According to Costanzo and Evola [113], Jamei and Chau [114], and Odli and Zakarya [115], the cooling effects provided by rooftop and vertical gardens mitigate UHI-induced dramatic temperature increases. In addition, rooftop and vertical gardens trap atmospheric pollutants, thus improving air quality [108,116].

In Japan’s capital Tokyo, several UHI mitigation measures have been implemented, including green premises, green roofs, green building walls, enhanced rooftop reflectivity, water-retentive pavement, and reductions in heat loss from buildings [117]. In Hong Kong, UHI mitigation strategies have primarily focused on preserving vegetation, improving ventilation routes, and reducing heat load in built-up regions by regulating urban morphology factors [118,119].

The second highest factor loading (0.887) belonged to SS5 (traffic congestion). According to [39], increased vehicular traffic and road congestion exacerbate the UHI situation and air pollution. Increased vehicular traffic also results in increased energy consumption, elevated emissions of greenhouse gases, and compromised human health and comfort [120]. Simpson [121] attributed a surge in vehicular traffic to the affordability and ease of ownership of private cars, as well as inefficiency of public transport.

### 3.5. Confirmatory Factor Analysis of UHI Adaptive Capacity Indicators

The UHI adaptive capacity component comprises three dimensions linked to three pillars of sustainability: social, economic and environment. The social, economic, and environment dimensions consist of four, four, and three UHI adaptive capacity indicators, respectively. The 11 UHI adaptive capacity indicators were validated with CFA to determine their factor loadings and reliability (first-order) and their dimensions (second-order), and SEM was used to analyze the relationships between the UHI indicators.

In the context of climate change, adaptive capacity refers to the ability of individuals to adjust to climate variability and extremes in order to mitigate potential impacts and cope with the consequences [122]. According to Abrar, Sarkar [123] UHI adaptive capacity refers to individuals’ capability to adapt to increased UHI exposure. 

**Figure 5:**
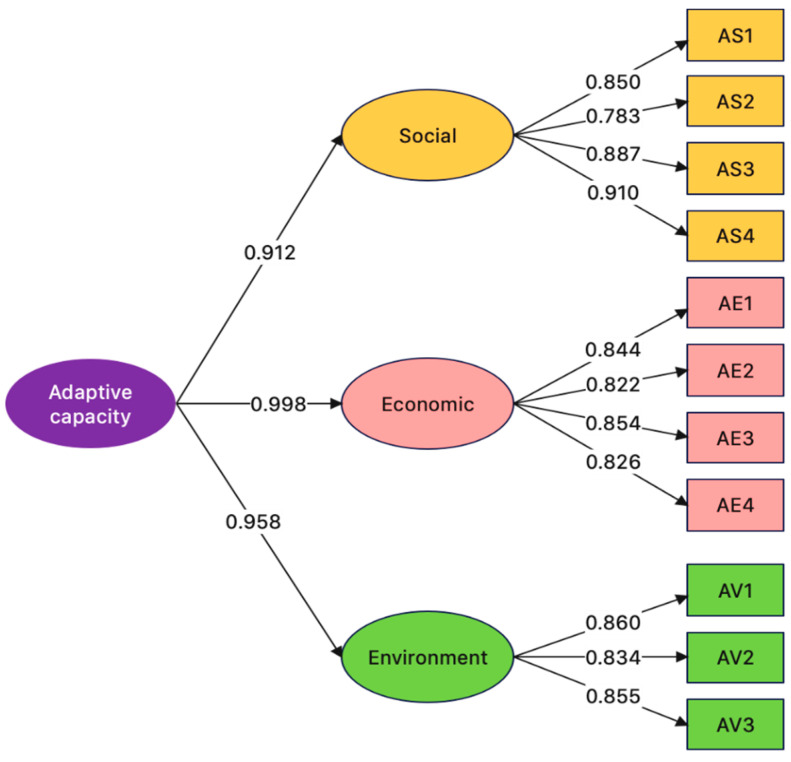
Structural equation model and the factor loadings of UHI adaptive capacity indicators and dimensions.

Figure 5 shows the SEM of the UHI adaptive capacity indicators, where chi-squared = 4092.786, df = 55, *p* = 0.109, RMR = 0.034, RMSEA = 0.039, GFI = 0.994, AGFI = 0.952, NFI = 0.996 and CFI = 0.999. 

Table 8 tabulates the first- and second-order factor loadings of the UHI adaptive capacity indicators and dimensions. The factor loadings of AS1–AS4, AE1–AE4 and AV1–AV3 were 0.783–0.910 (R2 = 0.613–0.828), 0.822–0.854 (R2 = 0.639–0.729), and 0.834–0.860 (R2 = 0.696–0.739), respectively, while those of the social, economic, and environment dimensions (b) were 0.912, 0.998, and 0.958, respectively; a factor loading >0.3 is statistically significant (Kim and Mueller [83]. The CR values of the social, economic, and environment dimensions were 0.918, 0.903, and 0.886, respectively with corresponding AVE values of 0.738, 0.670, and 0.722. According to Fornell and Larcker [84], a CFA construct is valid if CR >0.6 or AVE > 0.5.

The highest factor loading (0.910) belonged to AS4 (government policy and action), followed by AS3 (multi-agency collaboration) with a factor loading of 0.887. According to [58,122,123,124,125,126], government policy and action is crucial to the UHI adaptive capacity of urban residents. 

In addition, multi-agency collaboration on the dissemination of information and UHI impact mitigation enhances adaptive capacity to abrupt and dramatic temperature increases [127,128,129,130], Pineo and Zimmermann [131], and Smith and Levermore [69,132,133].

## 4. Conclusions

This research proposes a collection of 46 UHI risk indicators under four UHI risk components: hazard, exposure, sensitivity, and adaptive capacity. The UHI risk indicators were first validated by experts to determine their relevancy. The expert-validated UHI indicators were subsequently converted into a Likert-scale questionnaire and applied to randomly sampled residents of Thailand’s capital Bangkok. The respondents’ answers were input into SEM analysis.

In the SEM analysis, the 46 UHI risk indicators and four UHI risk components were validated with CFA to determine the factor loadings and reliability of the UHI risk indicators and dimensions. The indicator- and dimension-level factor loadings (0–1) were used to indicate the degree of relevancy of the UHI risk indicators and dimensions. 

In the UHI hazard component, the CFA factor loadings of the duration and temperature dimensions were 0.991 and 0.937, respectively, indicating that the duration dimension plays a slightly more significant role in the UHI hazard component than the temperature dimension. Furthermore, the highest indicator-level factor loading (0.915) belonged to HT6 (percentage of days when the daily minimum temperature is less than the 10th percentile, TN10).

In the UHI exposure component, the highest indicator-level CFA factor loading (0.923) belonged to ES4 (vehicular traffic). Vehicular traffic is the aggregation of vehicles coming and going in a particular locality and was positively correlated with UHI intensity. The second highest factor loading (0.917) belonged to EV3 (pervious surface area). The conversion of pervious to impervious surfaces as a result of economic development and urbanization expansion causes temperatures in urban areas to be significantly warmer than surrounding rural areas. 

Under the UHI sensitivity component, the proportion of green space to build environment indicator (SV1) had the highest factor loading (0.910). Urban green space provides cool and shaded areas while moderating ambient temperatures. Specifically, the UHI impacts were found to be inversely correlated with the proportion of urban green space to build environment. The second highest factor loading (0.887) belonged to SS5 (traffic congestion). Increased vehicular traffic and road congestion exacerbate the UHI situation and air pollution. Increased vehicular traffic also results in increased energy consumption, elevated emissions of greenhouse gases, and compromised human health and comfort.

In the UHI adaptive capacity component, the highest factor loading (0.910) belonged to AS4 (government policy and action), followed by AS3 (multi-agency collaboration) with a factor loading of 0.887. Government policy and action plays a crucial role in the UHI adaptive capacity of urban residents. In addition, multi-agency collaboration on the dissemination of information and UHI impact mitigation enhances adaptive capacity to abrupt and dramatic temperature increases.

In the implementation of the proposed statistically validated risk indicators to assess area-specific UHI susceptibility, the UHI indicators with high or very high factor loadings are converted into a questionnaire (i.e., UHI risk assessment tool) for primary data collection. In the event that the data are publicly available, the information related to the UHI indicators with high or very high factor loadings can be gathered from secondary sources (e.g., textbooks, reports, and news releases). The collected data (both primary and secondary data) are analyzed using normalized scores, percentages, or weighted average indexes to obtain area-specific UHI susceptibility index scores. The UHI susceptibility index scores are subsequently transformed into a UHI risk map that can be used by policymakers to formulate proper UHI mitigation measures.

### Research Implications

Although the proposed UHI indicators could be applied to assess UHI susceptibility, UHI risk assessment was outside the scope of this current research given that the aim of this research was to propose the statistically validated UHI risk indicators under four UHI risk components: hazard, exposure, sensitivity, and adaptive capacity. Nevertheless, the UHI risk assessment of 50 districts of metropolitan Bangkok will be carried out in subsequent research. 

In addition, since this current research was limited to one metropolitan area (i.e., Thailand’s capital Bangkok), future research could expand the study area to encompass diverse geographical locations, i.e., other major capital cities. Furthermore, in UHI risk assessment, the proposed 46 UHI risk indicators could be partially (<46 indicators) or wholly adopted, depending on data availability and the characteristics of the urban area.

## Figures and Tables

**Table 5:** First- and second-order CFA of UHI hazard indicators.

Dimension (Latent Factors)	CFA Construct Validity		UHI Hazard Indicators	Factor Loading	R^2^
	Composite Reliability (CR)	Average Variance Extracted (AVE)			
Temperature (b = 0.937)	0.921	0.660	HT1	0.806	0.682
			HT2	0.757	0.677
			HT3	0.785	0.660
			HT4	0.785	0.617
			HT5	0.818	0.670
			HT6	0.915	0.837
Duration (b = 0.991)	0.932	0.663	HD1	0.764	0.583
			HD2	0.821	0.674
			HD3	0.888	0.788
			HD4	0.840	0.706
			HD5	0.784	0.615
			HD6	0.822	0.675
			HD7	0.771	0.594

b is the factor loading of the second-order CFA. R^2^ is the reliability of the indicators.

**Table 6:** First- and second-order CFA of UHI exposure indicators.

Dimension (Latent Factors)	CFA Construct Validity		UHI Exposure Indicators	Factor Loading	R^2^
	Composite Reliability (CR)	Average Variance Extracted (AVE)			
Social (b = 0.997)	0.930	0.770	ES1	0.860	0.739
			ES2	0.864	0.747
			ES3	0.860	0.739
			ES4	0.923	0.853
Economic (b = 0.999)	0.887	0.724	EE1	0.808	0.683
			EE2	0.908	0.825
			EE3	0.833	0.695
Environment (b = 0.994)	0.925	0.804	EV1	0.894	0.799
			EV2	0.879	0.772
			EV3	0.917	0.840

b is the factor loading of the second-order CFA. R^2^ is the reliability of indicators.

**Table 7:** First- and second-order CFA of UHI sensitivity indicators.

Dimension (Latent Factors)	CFA Construct Validity		UHI Sensitivity Indicators	Factor Loading	R^2^
	Composite Reliability (CR)	Average Variance Extracted (AVE)			
Social (b = 0.936)	0.915	0.684	SS1	0.852	0.727
			SS2	0.839	0.703
			SS3	0.804	0.647
			SS4	0.745	0.500
			SS5	0.887	0.658
Economic (b = 0.981)	0.876	0.702	SE1	0.790	0.582
			SE2	0.855	0.731
			SE3	0.867	0.751
Environment (b = 0.999)	0.982	0.765	SV1	0.910	0.827
			SV2	0.846	0.721
			SV3	0.865	0.749
			SV4	0.876	0.767

b is the factor loading of the second-order CFA. R^2^ is the reliability of indicators.

**Table 8:** First- and second-order CFA of UHI adaptive capacity indicators.

Dimension (Latent Factors)	CFA Construct Validity		UHI Adaptive Capacity Indicators	Factor Loading	R^2^
	Composite Reliability (CR)	Average Variance Extracted (AVE)			
Social (b = 0.912)	0.918	0.738	AS1	0.850	0.723
			AS2	0.783	0.613
			AS3	0.887	0.787
			AS4	0.910	0.828
Economic (b = 0.998)	0.903	0.670	AE1	0.844	0.685
			AE2	0.822	0.639
			AE3	0.854	0.729
			AE4	0.826	0.724
Environment (b = 0.958)	0.886	0.722	AV1	0.860	0.739
			AV2	0.834	0.696
			AV3	0.855	0.730

b is the factor loading of the second-order CFA. R^2^ is the reliability of indicators.

## Data Availability

No new data were created.

## Supplementary Materials

The following supporting information can be downloaded at: https://www.mdpi.com/article/10.3390/ijerph20021172/s1.
