# Supplementary material for: Statistically Validated Urban Heat Island Risk Indicators for UHI Susceptibility Assessment
Source: Int J Environ Res Public Health. 2023 Jan 9;20(2):1172. doi: 10.3390/ijerph20021172 (PMC9859186; doi:10.3390/ijerph20021172)
Supplement: Supplementary file 1 [file ijerph-20-01172-s001.zip › ijerph-2046793-supplementary.pdf]

Supplementary Materials S1: Relevant/Irrelevant Questionnaire

The relevant/irrelevant questionnaire is electronically mailed to a panel of experts. The questionnaire asks the experts to validate the relevancy of the 46 UHI risk indictors under four UHI risk components: hazard, exposure, sensitivity, and adaptive capacity. The experts are provided with three choices: relevant, uncertain, and irrelevant. The responses of relevant, uncertain, and irrelevant are converted into item-objective congruence (IOC) indexes of 1, 0, and -1, respectively.

What is your current profession? (You can choose more than one option.)

- ☐ Urban environmentalist
- ☐ Healthcare personnel
- ☐ Urban planner
- ☐ Climate change scientist
- ☐ Architect
- ☐ Academic
- ☐ Other (please specify) ....

Component S1: Hazard Indicators (13 indicators)

Indicator	Definition/Detail	IOC		
		**Relevant (1)**	**Uncertain (0)**	**Irrelevant (-1)**
1	Cold spell duration index (CSDI). CSDI is defined as annual or seasonal count of days with at least 6 consecutive days when the daily minimum temperature falls below the 10th percentile in the calendar 5-day window for a 30-year period.			
2	Daily temperature range			
3	Monthly maximum value of daily maximum temperature			
4	Monthly maximum value of daily minimum temperature			
5	Monthly minimum value of daily maximum temperature			
6	Monthly minimum value of daily minimum temperature			
7	Number of summer days			
8	Number of tropical nights			
9	Percentage of days when daily maximum temperature is greater than the 90th percentile			
10	Percentage of days when daily maximum temperature is less than the 10th percentile			
11	Percentage of days when daily minimum temperature is greater than the 90th percentile			
12	Percentage of days when daily minimum temperature is less than the 10th percentile (TN10p)			
13	Warm spell duration index (WSDI). WSDI is defined as annual or seasonal count of days with at least 6 consecutive days when the daily maximum temperature exceeds the 90th percentile in the calendar 5-day window for a 30-year period.			

Component S2: Exposure Indicators (10 indicators)

Indicator	Definition/Detail	IOC		
		**Relevant (1)**	**Uncertain (0)**	**Irrelevant (-1)**
1	A metropolis with a population over one million has an average annual temperature of 1–3 °C higher than the surrounding rural areas.			
2	Electricity use is positively correlated to UHI. Specifically, electricity consumption and land surface temperature are strongly correlated with an R^2 of 70 – 90%.			
3	UHI contributes to heat-related deaths and illnesses such as general discomfort, respiratory difficulties, heat cramps, heat exhaustion, and non-fatal heat stroke.			

Indicator	Definition/Detail	IOC		
		Relevant (1)	Uncertain (0)	Irrelevant (-1)
	Sensitive populations are particularly at risk during excessive heat events, including the elderly, young children, those working outdoors, and those with preexisting health conditions.			
4	Vehicular traffic is the aggregation of vehicles coming and going in a particular locality. Vehicular traffic is positively correlated to the UHI intensity and air pollution.			
5	UHI is significantly positively correlated to population size ($p \leq 0.01$), economic size ($p \leq 0.01$), and urbanization ($p \leq 0.05$).			
6	The conversion of agricultural areas into commercial and industrial areas contributes to UHI. Urbanization expansion and increased human activities also worsen the UHI.			
7	Urbanization and economic growth contribute to population growth and increased economic activities, exacerbating the UHI phenomenon.			
8	Land surface temperatures vary in tandem with ambient temperatures. Increasing urban green space lowers land surface temperatures, which in turn reduces the ambient air temperatures.			
9	Impervious cover is any type of human-made surface that cannot effectively absorb or infiltrate rainfall, such as driveways, paved roads, parking lots, rooftops, and sidewalks. The expansion of impervious surface area contributes to worsening UHI problems.			
10	*Pervious surface* is land not covered by buildings or other man-made infrastructure, thus allowing rainwater to percolate into the soil to filter out pollutants and recharge the groundwater. The pervious surface coverage and UHI are inversely related.			

Component S3: Sensitivity Indicators (12 indicators)

Indicator	Definition/Detail	IOC		
		Relevant (1)	Uncertain (0)	Irrelevant (-1)
1	Population density and growth is closely linked to human activities and UHI. A larger population contributes to higher greenhouse gas emissions as a result of increased vehicular traffic and electricity consumption, giving rise to dramatic temperature increases.			
2	The built environment touches all aspects of human lives, including buildings, public utilities and infrastructure. Specifically, the conversion of green area into built environment contributes to UHI and the situation worsens as the conversion intensifies.			
3	Total energy consumption is the sum of energy used for electricity, transport and heating. Total energy consumption is positively correlated to human activities, which in turn contributes to the UHI effects.			
4	Noncommunicable diseases (NCD) are of long duration and are the result of a combination of genetic, physiological, environmental and behavioral factors. Examples of NCD are cardiovascular disease, diabetes, high blood pressure, obesity.<br><br>Individuals with NCD or preexisting health conditions are highly susceptible to UHI-related excessive heat events.			
5	Increased vehicular traffic and road congestion exacerbates the UHI situation and air pollution.			
6	Individuals with low income are highly susceptible to the UHI impacts. Specifically, low-income households have inadequate income or wealth to mitigate the negative impacts of UHI-induced excessive heat events.			

Indicator	Definition/Detail	IOC		
		Relevant (1)	Uncertain (0)	Irrelevant (-1)
7	Monthly household electricity cost is in direct proportion to electricity consumption. Higher electricity consumption in turn aggravates the UHI situation.			
8	Higher monthly household energy (motor fuels) spending is in direct proportion to increased vehicle use, which in turn contributes to UHI and air pollution.			
9	Green space provides cool and shaded areas while moderating ambient temperatures. The proportion of green space to man-made built environment is inversely correlated to UHI.			
10	Anthropogenic atmospheric pollutants contribute to rising temperatures and UHI. The UHI-causing atmospheric pollutants include CO, NO_2, O_3, PM2.5, and PM10.			
11	Temperatures in and near water body are significantly lower than those covered by built environment. Besides, the proportion of water body to man-made built environment is inversely correlated to UHI.			
12	Building density (measured by dwelling units per km^2) determines how crowded or built-up an area of land is.<br>An area with high building density exhibits high UHI intensity.			

Component S4: Adaptive Capacity Indicators (11 indicators)

Indicator	Definition/Detail	IOC		
		Relevant (1)	Uncertain (0)	Irrelevant (-1)
1	Public understanding of UHI and its impacts enhances adaptive capacity to excessive heat events or UHI-induced temperature increases.			
2	Greater public awareness of UHI and its risks contributes to increased UHI adaptive capacity and resilience.			
3	Multi-agency collaboration on dissemination of information and UHI impact mitigation enhances adaptive capacity to abrupt and dramatic temperature increases.			
4	Governments (i.e., local, regional and national levels) should put in place UHI mitigation policy and measures to enhance adaptive capacity to UHI-induced excessive heat events.			
5	Green budgeting refers to the adoption of budgetary tools to achieve environmental and climate goals. Higher green budget allocation enhances adaptive capacity to UHI.			
6	A green economy is an economy that aims at reducing environmental risks and ecological scarcities while achieving sustainable development without degrading the environment. Specifically, active participation of the private sector in green economy enhances adaptive capacity to UHI-related dramatic temperature increases.			
7	Access to climate control appliances, e.g., electric fan, cooling fan, air conditioner, increases adaptive capacity to UHI-induced excessive heat events.			
8	The size of household medical budget is positively correlated to adaptive capacity to UHI-related illnesses.			
9	Green space is land that is partly or completely covered with grass, trees, shrubs, or other vegetation. Meanwhile, living space means areas in a dwelling unit that are livable space. Higher proportion of green space to living space improves adaptive capacity to UHI-induced temperature increases.			
10	Urban green space is open-space areas reserved for parks and recreational activities. Adequate urban green space improves adaptive capacity to UHI-related abrupt temperature increases.			
11	Individual resilience to atmospheric pollutants and extreme heat events enhances adaptive capacity to UHI-related illnesses and deaths.			

Supplementary Materials S2: Agree/Disagree Questionnaire

The agree/disagree questionnaire is applied to a random sample of Bangkok residents in 50 districts. The questionnaire asks the participants to validate (i.e., agree or disagree with) the relevancy of the 46 UHI risk indictors under the four UHI components, on a 10-point Likert scale, where 1 denotes strongly disagree and 10 strongly agree.

1) Your place of residency (please specify the Bangkok district where you reside) ....
2) Choose the following items that are applicable to you (You can choose more than one option.)

☐ Individual affected by UHI

☐ Permanent Bangkok resident

☐ Healthcare personnel

☐ Employed in the field of urban environment and/or climate change

☐ Employed in the field of urban planning and development

☐ None of the above.

Component S1: Hazard Indicators (13 indicators)

Indicator	Definition/Detail	1 denotes strongly disagree and 10 strongly agree									
		1	2	3	4	5	6	7	8	9	10
1	Cold spell duration index (CSDI). CSDI is defined as annual or seasonal count of days with at least 6 consecutive days when the daily minimum temperature falls below the 10th percentile in the calendar 5-day window for a 30-year period.										
2	Daily temperature range										
3	Monthly maximum value of daily maximum temperature										
4	Monthly maximum value of daily minimum temperature										
5	Monthly minimum value of daily maximum temperature										
6	Monthly minimum value of daily minimum temperature										
7	Number of summer days										
8	Number of tropical nights										
9	Percentage of days when daily maximum temperature is greater than the 90th percentile										
10	Percentage of days when daily maximum temperature is less than the 10th percentile										
11	Percentage of days when daily minimum temperature is greater than the 90th percentile										
12	Percentage of days when daily minimum temperature is less than the 10th percentile (TN10p)										
13	Warm spell duration index (WSDI). WSDI is defined as annual or seasonal count of days with at least 6 consecutive days when the daily maximum temperature exceeds the 90th percentile in the calendar 5-day window for a 30-year period.										

Component S2: Exposure Indicators (10 indicators)

Indicator	Definition/Detail	1 denotes strongly disagree and 10 strongly agree									
		1	2	3	4	5	6	7	8	9	10
1	A metropolis with a population over one million has an average annual temperature 1–3 °C higher than the surrounding rural areas.										
2	Electricity use is positively correlated to UHI. Specifically, electricity consumption and land										

Indicator	Definition/Detail	1 denotes strongly disagree and 10 strongly agree									
		1	2	3	4	5	6	7	8	9	10
	surface temperature are strongly correlated with an R^2 of 70 – 90%.										
3	UHI contributes to heat-related deaths and illnesses such as general discomfort, respiratory difficulties, heat cramps, heat exhaustion, and non-fatal heat stroke.<br><br>Sensitive populations are particularly at risk during excessive heat events, including the elderly, young children, those working outdoors, and those with preexisting health conditions.										
4	Vehicular traffic is the aggregation of vehicles coming and going in a particular locality. Vehicular traffic is positively correlated to the UHI intensity and air pollution.										
5	UHI is significantly positively correlated to population size ($p \leq 0.01$), economic size ($p \leq 0.01$), and urbanization ($p \leq 0.05$).										
6	The conversion of agricultural areas into commercial and industrial areas contributes to UHI. Urbanization expansion and increased human activities also worsen the UHI.										
7	Urbanization and economic growth contribute to population growth and increased economic activities, exacerbating the UHI phenomenon.										
8	Land surface temperatures vary in tandem with ambient temperatures. Increasing urban green space lowers land surface temperatures, which in turn reduces the ambient air temperatures.										
9	Impervious cover is any type of human-made surface that cannot effectively absorb or infiltrate rainfall, such as driveways, paved roads, parking lots, rooftops, and sidewalks. The expansion of impervious surface area contributes to worsening UHI problems.										
10	Pervious surface is land not covered by buildings or other man-made infrastructure, thus allowing rainwater to percolate into the soil to filter out pollutants and recharge the groundwater. The pervious surface coverage and UHI are inversely related.										

Component S3: Sensitivity Indicators (12 indicators)

Indicator	Definition/Detail	1 denotes strongly disagree and 10 strongly agree									
		1	2	3	4	5	6	7	8	9	10
1	Population density and growth is closely linked to human activities and UHI. A larger population contributes to higher greenhouse gas emissions as a result of increased vehicular traffic and electricity consumption, giving rise to dramatic temperature increases.										
2	The built environment touches all aspects of human lives, including buildings, public utilities and infrastructure. Specifically, the conversion of green area into built environment contributes to UHI and the situation worsens as the conversion intensifies.										
3	Total energy consumption is the sum of energy used for electricity, transport and heating. Total energy consumption is positively correlated to human activities, which in turn contributes to the UHI effects.										

Indicator	Definition/Detail	1 denotes strongly disagree and 10 strongly agree									
		1	2	3	4	5	6	7	8	9	10
4	Noncommunicable diseases (NCD) are of long duration and are the result of a combination of genetic, physiological, environmental and behavioral factors. Examples of NCD are cardiovascular disease, diabetes, high blood pressure, obesity.<br><br>Individuals with NCD or preexisting health conditions are highly susceptible to UHI-related excessive heat events.										
5	Increased vehicular traffic and road congestion exacerbates the UHI situation and air pollution.										
6	*Individuals with low income are highly susceptible to the UHI impacts. Specifically,* low-income households have inadequate income or wealth to mitigate the negative impacts of UHI-induced excessive heat events.										
7	Monthly household electricity cost is in direct proportion to electricity consumption. Higher electricity consumption in turn aggravates the UHI situation.										
8	Higher monthly household energy (motor fuels) spending is in direct proportion to increased vehicle use, which in turn contributes to UHI and air pollution.										
9	Green space provides cool and shaded areas while moderating ambient temperatures. The proportion of green space to man-made built environment is inversely correlated to UHI.										
10	Anthropogenic atmospheric pollutants contribute to rising temperatures and UHI. The UHI-causing atmospheric pollutants include CO, NO_2, O_3, PM2.5, and PM10.										
11	Temperatures in and near water body are significantly lower than those covered by built environment. Besides, the proportion of water body to man-made built environment is inversely correlated to UHI.										
12	Building density (measured by dwelling units per km^2) determines how crowded or built-up an area of land is.<br>An area with high building density exhibits high UHI intensity.										

Component S4: Adaptive Capacity Indicators (11 indicators)

Indicator	Definition/Detail	1 denotes strongly disagree and 10 strongly agree									
		1	2	3	4	5	6	7	8	9	10
1	Public understanding of UHI and its impacts enhances adaptive capacity to excessive heat events or UHI-induced temperature increases.										
2	Greater public awareness of UHI and its risks contributes to increased UHI adaptive capacity and resilience.										
3	Multi-agency collaboration on dissemination of information and UHI impact mitigation enhances adaptive capacity to abrupt and dramatic temperature increases.										
4	Governments (i.e., local, regional and national levels) should put in place UHI mitigation policy and measures to enhance adaptive capacity to UHI-induced excessive heat events.										

Indicator	Definition/Detail	1 denotes strongly disagree and 10 strongly agree									
		1	2	3	4	5	6	7	8	9	10
5	Green budgeting refers to the adoption of budgetary tools to achieve environmental and climate goals. Higher green budget allocation enhances adaptive capacity to UHI.										
6	A green economy is an economy that aims at reducing environmental risks and ecological scarcities while achieving sustainable development without degrading the environment. Specifically, active participation of the private sector in green economy enhances adaptive capacity to UHI-related dramatic temperature increases.										
7	Access to climate control appliances, e.g., electric fan, cooling fan, air conditioner, increases adaptive capacity to UHI-induced excessive heat events.										
8	The size of household medical budget is positively correlated to adaptive capacity to UHI-related illnesses.										
9	Green space is land that is partly or completely covered with grass, trees, shrubs, or other vegetation. Meanwhile, living space means areas in a dwelling unit that are livable space. Higher proportion of green space to living space improves adaptive capacity to UHI-induced temperature increases.										
10	Urban green space is open-space areas reserved for parks and recreational activities. Adequate urban green space improves adaptive capacity to UHI-related abrupt temperature increases.										
11	Individual resilience to atmospheric pollutants and extreme heat events enhances adaptive capacity to UHI-related illnesses and deaths.										
